# Ultra-low dose dual-layer detector spectral CT for pulmonary nodule screening: image quality and diagnostic performance

**DOI:** 10.1186/s13244-024-01888-1

**Published:** 2025-01-10

**Authors:** Li Ding, Mingwang Chen, Xiaomei Li, Yuting Wu, Jingxu Li, Shuting Deng, Yikai Xu, Zhao Chen, Chenggong Yan

**Affiliations:** 1 Department of Medical Imaging Center, Nanfang Hospital, Southern Medical University, Guangzhou, 510515 China; 2 Department of Radiology, The First Affiliated Hospital of Guangzhou Medical University, Guangzhou, 510120 China

**Keywords:** Pulmonary nodule, Ultra-low dose CT, Dual-layer detector CT, Image quality

## Abstract

**Objectives:**

To investigate the image quality and diagnostic performance with ultra-low dose dual-layer detector spectral CT (DLSCT) by various reconstruction techniques for evaluation of pulmonary nodules.

**Materials and methods:**

Between April 2023 and December 2023, patients with suspected pulmonary nodules were prospectively enrolled and underwent regular-dose chest CT (RDCT; 120 kVp/automatic tube current) and ultra-low dose CT (ULDCT; 100 kVp/10 mAs) on a DLSCT scanner. ULDCT was reconstructed with hybrid iterative reconstruction (HIR), electron density map (EDM), and virtual monoenergetic images at 40 keV and 70 keV. Quantitative and qualitative image analysis, nodule detectability, and Lung-RADS evaluation were compared using repeated one-way analysis of variance, Friedman test, and weighted kappa coefficient.

**Results:**

A total of 249 participants (mean age ± standard deviation, 50.0 years ± 12.9; 126 male) with 637 lung nodules were included. ULDCT resulted in a significantly lower mean radiation dose than RDCT (0.3 mSv ± 0.0 vs. 3.6 mSv ± 0.8; *p* < 0.001). Compared with RDCT, ULDCT EDM showed significantly higher signal-noise-ratio (44.0 ± 77.2 vs. 4.6 ± 6.6; *p* < 0.001) and contrast-noise-ratio (26.7 ± 17.7 vs. 5.0 ± 4.4; *p* < 0.001) with qualitative scores ranked higher or equal to the average. Using the regular-dose images as a reference, ULDCT EDM images had a satisfactory nodule detection rate (84.6%) and good inter-observer agreements compared with RDCT (κw > 0.60).

**Conclusion:**

Ultra-low dose dual-layer detector CT with 91.2% radiation dose reduction achieves sufficient image quality and diagnostic performance of pulmonary nodules.

**Critical relevance statement:**

Dual-layer detector spectral CT enables substantial radiation dose reduction without impairing image quality for the follow-up of pulmonary nodules or lung cancer screening.

**Key Points:**

- Radiation dose is a major concern for patients requiring pulmonary nodules CT screening.
- Ultra-low dose dual-layer detector spectral CT with 91.2% dose reduction demonstrated satisfactory performance.
- Dual-layer detector spectral CT has the potential for lung cancer screening and management.

**Graphical Abstract:**

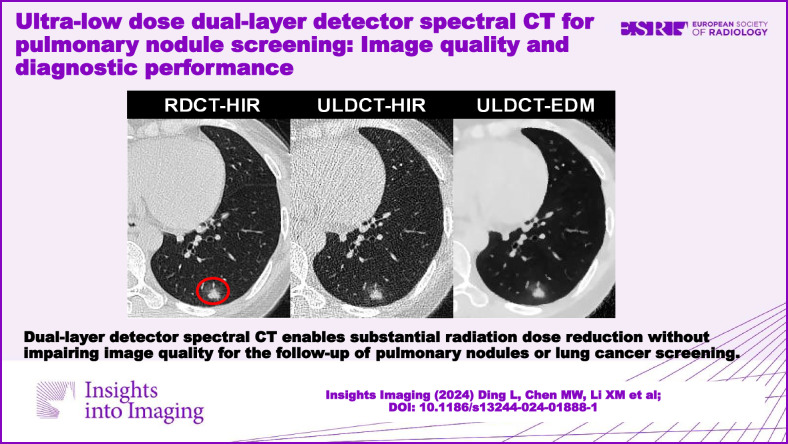

## Introduction

CT has been recommended as the standard diagnostic imaging modality for the evaluation of lung nodules. According to Lung Imaging Reporting and Data System (Lung-RADS) management recommendations [1], and large multi-center trials have demonstrated the value of CT for lung cancer screening in high-risk patients [2–5]. As the requirement of repeated long-term follow-up, concerns regarding radiation exposure have increased markedly over the past 20 years [6]. According to the principle of as low as reasonably achievable (ALARA), the radiation dose needs to be optimized to minimize cancer risk [7, 8]. However, radiation dose reduction while maintaining diagnostic quality remains a major challenge in clinical practice.

To prevent radiation-associated cancer risk, various strategies, including CT hardware and software technological optimization, have been explored, such as iterative reconstruction (IR) algorithms, tube current modulation, and automatic exposure control [9–15]. Recently, clinically used IR algorithms or deep learning networks have enabled a significant decrease in radiation dose without impairing image quality [11–15]. A prior study has reported that iterative model reconstruction images from ultra-low-dose CT (ULDCT), for which the effective dose (mean 0.14 mSv) is equivalent to a chest X-ray radiography, is feasible for evaluation of pulmonary invasive fungal infections with satisfactory spatial resolution [16]. However, model-based IR has been criticized for long reconstruction time (~15 min) and altered visual appearance (smoothing effect), which may limit its clinical application [17].

More recently, dual-layer detector spectral CT (DLSCT) based on sandwich detectors has been introduced for clinical use. Unlike conventional polyenergetic CT, DLSCT is believed to offer several advantages in comparison to conventional CT, e.g., a reduction of image noise and an improvement of the contrast-to-noise ratio [18–21]. Many studies have shown that virtual monoenergetic image (VMI) at a low-energy level is feasible to enhance lesion contrast on CT images [22–24]. Further technical advancements in spectral CT now allow measurement of electron density map (EDM), which describe promising results that could enhance the lung lesion extent assessment of ground-glass opacity in patients with early-stage COVID-19 or predict the invasiveness of lung adenocarcinoma manifesting as pure ground-glass nodules (GGNs) [25, 26]. This information may provide valuable insight regarding changes in electron composition in the pathologic tissue. However, to the best of our knowledge, the potential of the reduced radiation dose with DLSCT in the clinical routine has not been evaluated yet.

Therefore, the purpose of this study was to investigate the value of ultra-low dose DLSCT for evaluation of lung nodules and to compare the image quality and diagnostic performance with regular-dose CT (RDCT).

## Materials and methods

### Participants

**Fig. 1:**
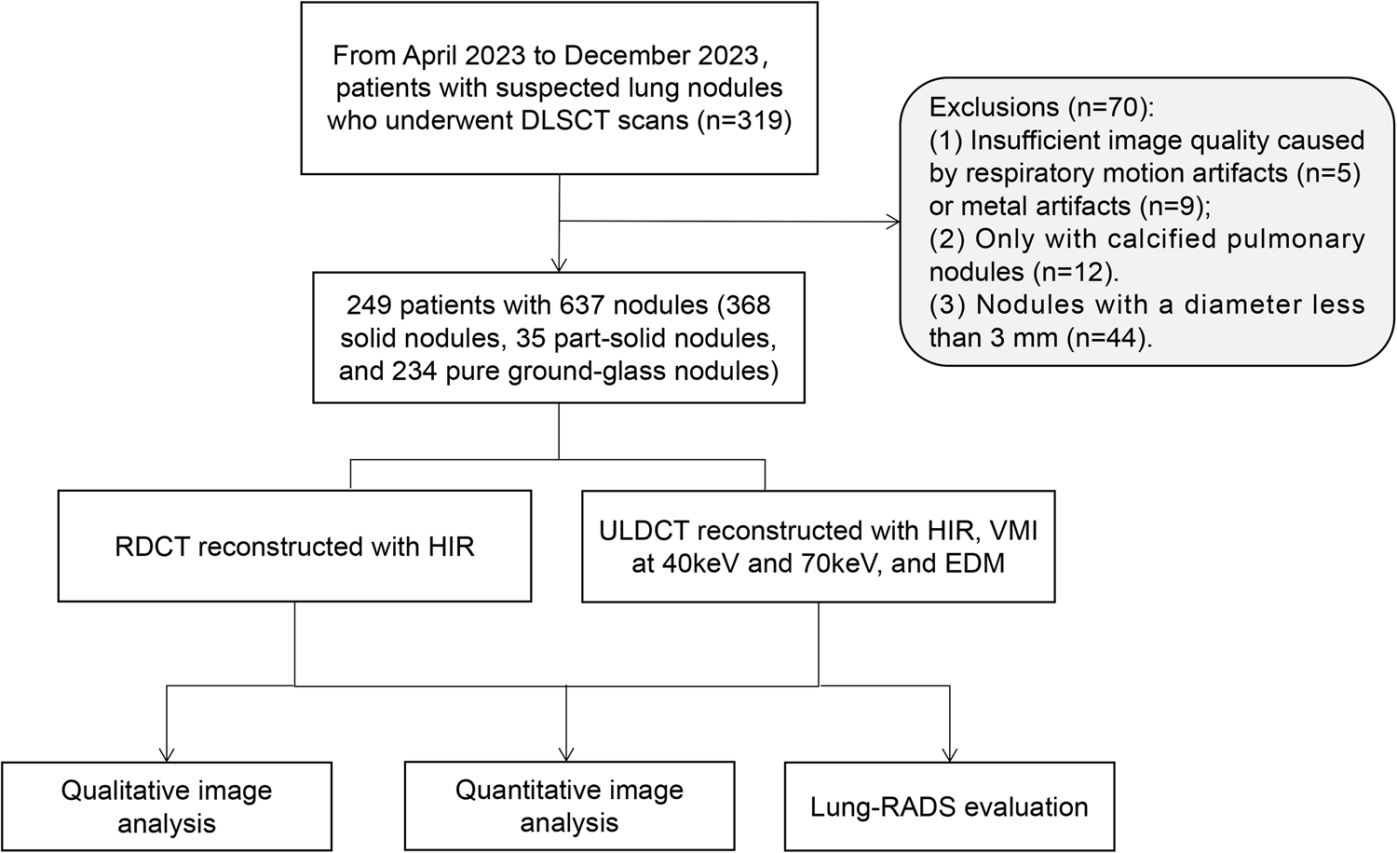
The inclusion and exclusion criteria of this study. DLSCT, dual-layer detector spectral CT; RDCT, regular-dose CT; ULDCT, ultra-low dose CT; HIR, hybrid iterative reconstruction; VMI, virtual monoenergetic image; EDM, electron density mapping; Lung-RADS, Lung Imaging Reporting and Data System

This prospective study was approved by our institutional review board, and written informed consent was obtained. From April 2023 to December 2023, patients with suspected lung nodules were enrolled. The inclusion criteria (Fig. 1) were as follows: (1) age over 18 years old, and (2) lung cancer screening or routine follow-up of previously identified lung nodule(s). Exclusion criteria were (1) insufficient image quality caused by respiratory motion artifacts (*n* = 5) or metal artifacts (*n* = 9), (2) only with calcified pulmonary nodules (*n* = 12), and (3) nodules with a diameter less-than 3 mm (*n* = 44).

### CT protocol and image reconstruction

All CT examinations were performed using a DLSCT scanner (Hawk, Philips Healthcare, the Netherlands). Each patient underwent an unenhanced RDCT scan first, followed by a ULDCT scan. The detailed scanning parameters are provided in the Supplementary Materials (Appendix E1). A total of five series of CT scans (regular-dose with hybrid IR (HIR), ultra-low dose with HIR, and ultra-low dose spectral data reconstructed with EDM, VMI at 40 keV and 70 keV) were obtained. All images were reconstructed with a slice thickness of 1.0 mm and lung window settings (window width, 1600 HU; window center, −600 HU).

### Radiation dose

The volume CT dose index (CTDI_vol_, mGy) and dose-length product (DLP, mGy·cm) were recorded for ULDCT and RDCT. Effective dose (ED) in mSv was calculated as DLP multiplied by a *k*-factor of 0.014 mSv/mGy·cm [27].

### Qualitative image analysis

Two thoracic radiologists (L.D. and M.C., with 6 and 3 years of experience), who were blinded to scanning parameters and reconstruction techniques, reviewed the image series independently on a post-processing workstation (IntelliSpace Portal, Philips Healthcare). All CT scans were anonymous and displayed in random order with a lung window setting, allowing readers to adjust. Streak artifact, image noise, clarity of small vessels, visibility of nodules, and the overall image quality were subjectively graded with 5-point scales (Table 1). If there was any discrepancy between the two observers, further analysis was performed by a third experienced radiologist (C.Y., with 15 years of experience in radiology) to reach a consensus. To standardize grading criteria for image quality, the radiologists were individually trained with 10 image datasets which were not included in this study, before the reading session.

**Table 1:** Evaluation criteria for qualitative image quality

Score	Streak artifact	Image noise	Clarity of small vessels	Visibility of pulmonary nodules	Overall image quality
1	Very poor	Unacceptable noise	Unclear edge	Not distinct	Very poor, cannot distinguish small structures
2	Suboptimal	Above-average increased noise	Fuzzy edge	Barely distinct	Suboptimal, small structures are not displayed very well
3	Average	Average noise in an acceptable image	Not very clear edge	Moderately distinct	Moderate, small structures can be displayed
4	Better than average	Less-than average noise	Still clear edge	Fairly distinct	Better than average, small structures can be clearly displayed with good contrast
5	Excellent	Minimum or no image noise	Clear edge	Definitely distinct	Excellent, small structures can be clearly displayed with excellent contrast

### Quantitative image analysis

Quantitative analysis was performed by one thoracic radiologist (L.D.) to evaluate the CT number, noise, signal-to-noise ratio (SNR), and contrast-to-noise ratio (CNR). Circular regions of interest (ROIs) were manually placed centrally in subcutaneous fat, paravertebral muscles, and thoracic descending aorta at the level of the pulmonary trunk or in nodules at the largest area on the transverse section. The largest possible ROIs were drawn while avoiding neighboring normal anatomic structures, calcification, and necrotic areas. The mean CT attenuation values (in Hounsfield Units) or water content percentage, along with the standard deviation (SD), were measured on each reconstruction. The SD for each anatomic structure in ROI was defined as image noise. SNRs and CNRs were calculated using the following formulas: SNR = CT_ROI_/SD_ROI_ and CNR = (CT_fat_ − CT_aorta_)/SD_aorta_, respectively.

### Nodule detection and reference standard

The RDCT images were imported into a commercially available artificial intelligence (AI) software Deepwise^TM^, which was based on deep neural networks. All nodules were identified by the AI software and then visually confirmed by a principal investigator (L.D.), and the final results were considered as the reference standard. Nodules with a diameter less-than 3 mm were excluded from our study, due to measurement difficulty in practice. The diameter, volume, type (i.e., solid, part-solid, or pure ground-glass), and Lung-RADS category of each nodule were recorded.

The diameter and volume of nodules on ULDCT images were evaluated by the same radiologist blinded to all information, with a delay period of 3 weeks to minimize recall bias. Thirty nodules were randomly selected for evaluation of diameter and volume measurement consistency by another radiologist (X.L.). The nodule detection rate and Lung-RADS classification on different reconstruction images were assessed by two readers (L.D. and X.L.) on the post-processing workstation independently. All measurements were performed with the semi-automated Lung Nodule Assessment software (IntelliSpace Portal, Philips Healthcare). Nodules that could not be identified in the evaluation images were considered false negatives.

### Statistical analysis

Statistical analyses were performed by using SPSS version 22.0 (IBM Corp, Armonk, NY, USA). Kolmogorov–Smirnov test was used to examine the data normality. A repeated one-way analysis of variance (ANOVA) was performed to compare the quantitative image quality measurements, diameter, and volume, with a Bonferroni post hoc test for multiple comparisons. Bland-Altman analyses and intraclass correlation coefficients (ICCs) were used to compare the diameter and volume measured correlation between RDCT and ULDCT. Qualitative image quality assessment was compared by using the Friedman test and post hoc Wilcoxon signed-rank test. A weighted kappa coefficient (κw) was used for the inter-scan and inter-observer agreement of Lung-RADS categories per reconstructions or radiologists. The radiation dose between the RDCT and ULDCT was compared using a paired *t*-test. A *p*-value of < 0.05 and a Bonferroni-corrected *p-*value of < 0.0125 (0.05/4) were considered statistically significant. Agreement on the basis of ICC or weighted kappa values was classified as follows: poor (< 0.20), fair (0.20–0.40), moderate (0.40–0.60), good (0.60–0.80), or excellent (> 0.80).

## Results

### Patient demographics

A total of 249 patients (126 male, 123 female) with a mean age of 50.0 ± 12.9 years (range, 19–78 years) were included. The mean body mass index (BMI) was 22.9 ± 3.0 kg/m^2^ (range, 12.5–32.6 kg/m^2^), with 16 patients with BMI < 18.5 kg/m^2^ and 91 patients with BMI ≥ 24 kg/m^2^. Patient characteristics are summarized in Table 2.

**Table 2:** Patient characteristics

Characteristics	Patients (*n* = 249)
Sex (men: women)	126: 123
Age (years)^a^	50.0 ± 12.9
Body mass index (kg/m^2^)^a^	22.9 ± 3.0
< 18.5/18.5–24/ ≥ 24	16: 142: 91
All nodules	637
Components (solid: part-solid: pure ground-glass)	368: 35: 234
Lung-RADS category (1: 2: 3: 4A: 4B: 4X)	56: 394: 136: 30: 13: 8
Solid nodules Lung-RADS category (1: 2: 3: 4A: 4B: 4X)	56: 160: 110: 30: 7: 5
Part-solid nodules Lung-RADS category (3: 4A: 4B: 4X)	26: 0: 6: 3
Pure ground-glass nodules Lung-RADS category (2)	234
Mean diameter of transverse(mm)	6.0 ± 3.2
Volume (cm^3^)^a^	260.1 ± 844.2

^a^ Data are shown as means ± standard deviation

According to the reference standard, a total of 637 lung nodules were identified. Among them, 368 (57.8%) were solid, 35 (5.5%) were part-solid, and 234 (36.7%) were pure ground-glass. The nodules were divided according to the type (solid, part-solid, and pure ground-glass) and Lung-RADS category for subgroup analysis.

### Radiation dose

The mean CTDI_vol_ (0.5 mGy × cm ± 0.0 vs. 5.6 ± 1.2 mGy × cm; *p* < 0.001) and dose-length product (23.0 mGy ×cm ± 1.4 vs. 258.4 mGy × cm ± 54.4; *p* < 0.001) were significantly lower with ULDCT than with RDCT. The mean effective dose of RDCT and ULDCT was 3.6 mSv ± 0.8 and 0.3 mSv ± 0.0, respectively, indicating a significant dose reduction of 91.2% (*p* < .001).

### Qualitative image analysis

**Fig. 2:**
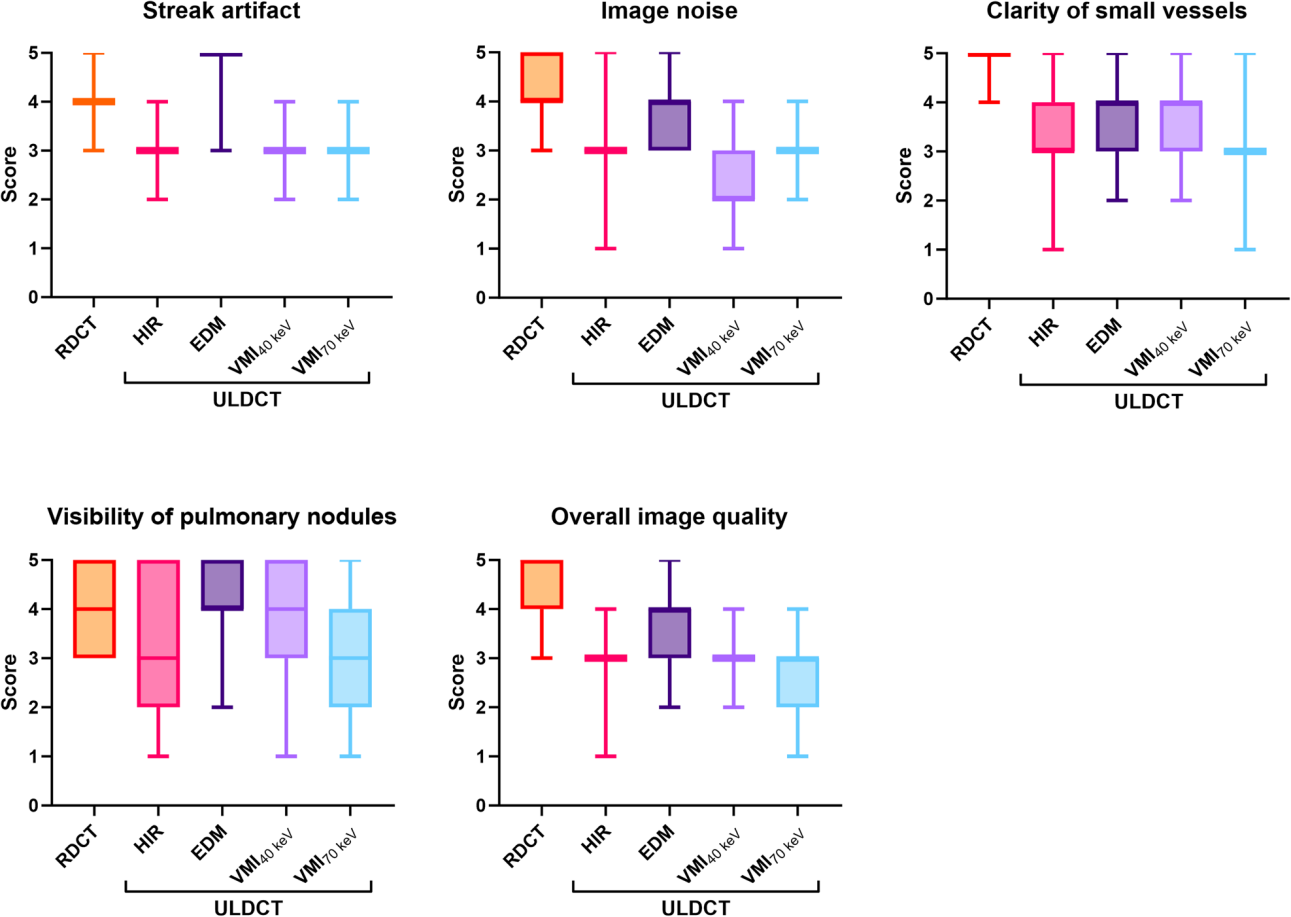
The box plots show the qualitative image quality scoring results. For visibility of pulmonary nodules, no statistically significant difference is found between RDCT HIR and ULDCT EDM (*p* > 0.05). And significant differences are observed among other reconstruction groups (*p * < 0.001 for all). DLSCT, dual-layer detector spectral CT; RDCT, regular-dose CT; ULDCT, ultra-low dose CT; HIR, hybrid iterative reconstruction; VMI, virtual monoenergetic image; EDM, electron density mapping

Figure 2 shows box plots of the qualitative scoring results. For ULDCT images, all image quality parameters were ranked superior with EDM when compared with HIR and VMIs (*p* < 0.05 for all comparisons). Notably, the average scores of ULDCT EDM in each assessment item were higher or equal to the average (score 3 on a 5-point scale). The inter-observer agreement (kappa value) for streak artifact, image noise, clarity of small vessels, visibility of nodules, and the overall image quality were 0.75, 0.70, 0.71, 0.74, and 0.68, respectively.

**Fig. 3:**
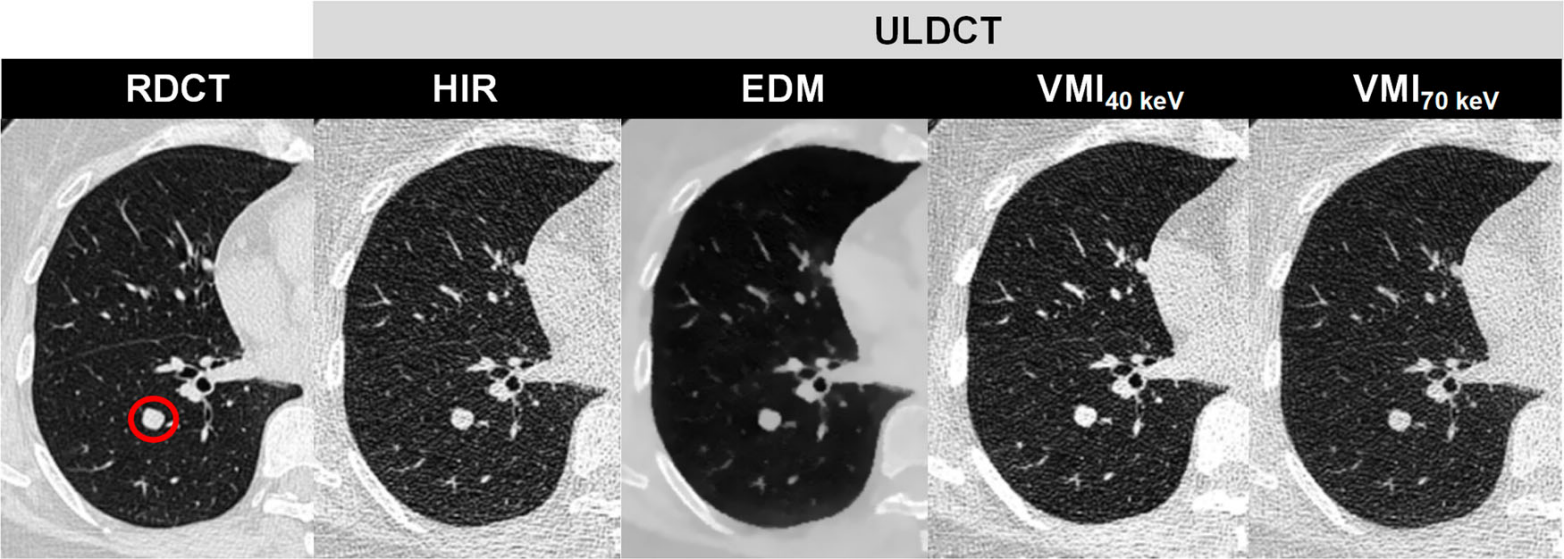
DLSCT at different radiation doses and reconstruction algorithms in a 65-year-old female (BMI: 21.2 kg/m^2^) with a solid nodule in the right lower lobe (circle). RDCT, regular-dose CT; ULDCT, ultra-low dose CT; HIR, hybrid iterative reconstruction; VMI, virtual monoenergetic image; EDM, electron density mapping

**Fig. 4:**
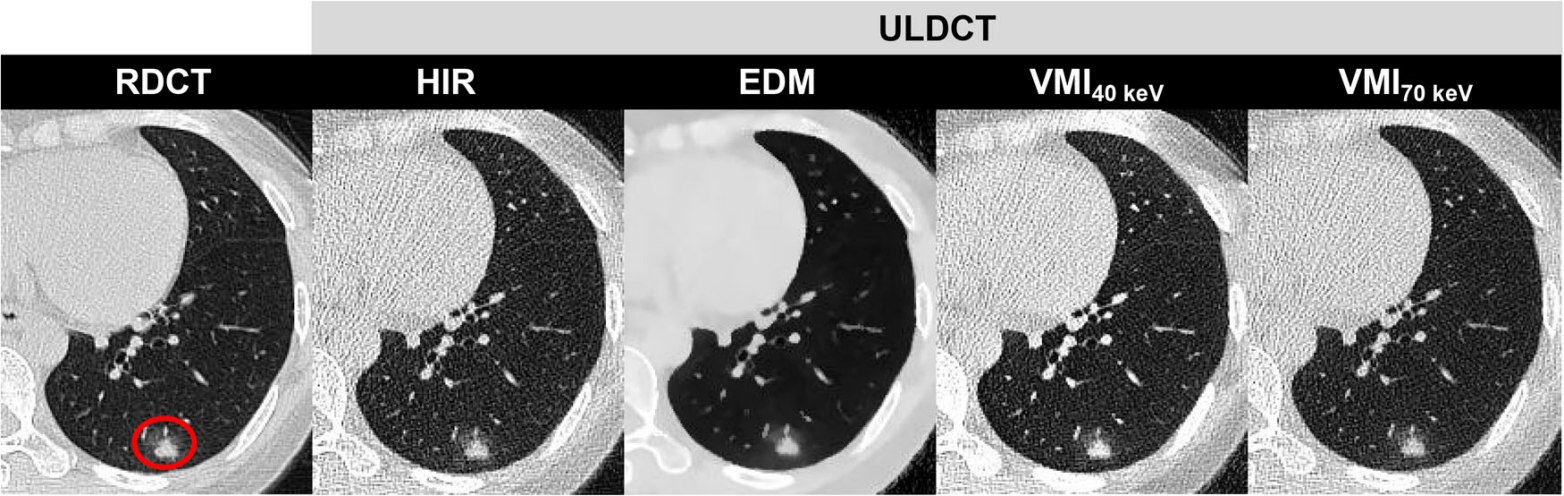
DLSCT at different radiation doses and reconstruction algorithms in a 49-year-old female (BMI: 19.5 kg/m^2^) with a part-solid nodule in the left lower lobe (circle). RDCT, regular-dose CT; ULDCT, ultra-low dose CT; HIR, hybrid iterative reconstruction; VMI, virtual monoenergetic image; EDM, electron density mapping

**Fig. 5:**
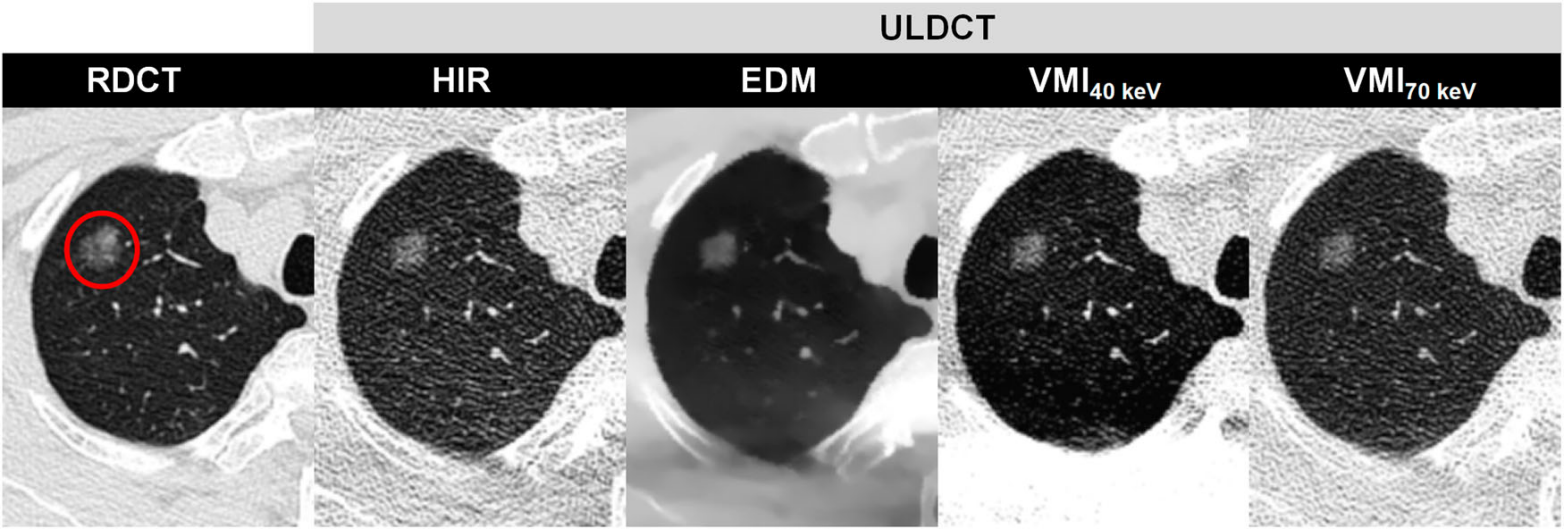
DLSCT at different radiation doses and reconstruction algorithms in a 67-year-old male (BMI: 25.4 kg/m^2^) with a pure ground-glass nodule in the right upper lobe (circle). RDCT, regular-dose CT; ULDCT, ultra-low dose CT; HIR, hybrid iterative reconstruction; VMI, virtual monoenergetic image; EDM, electron density mapping

In detail, overall image quality for ULDCT EDM was rated as excellent in 15.5% (99 of 637), good in 48.8% (311 of 637), and moderate in 34.2% (218 of 637). In terms of the visibility of all pulmonary nodules, the difference was not significant between RDCT HIR and ULDCT EDM (4.4 ± 0.8 vs. 4.3 ± 0.8; *p* > 0.05). For pure GGN, ULDCT EDM scored significantly better than RDCT HIR (4.0 ± 0.8 vs. 3.6 ± 0.7; *p* < 0.001). For part-solid nodules, a similar result was found (ULDCT EDM 4.8 ± 0.4 vs. RDCT HIR 4.7 ± 0.6, *p* > 0.05). For solid nodules, RDCT HIR achieved the highest score (4.5 ± 0.7), and EDM and VMI_40 keV_ on ULDCT were of diagnostic quality (score > 3). Examples of RDCT and ULDCT images in participants were provided in Figs. 3–5. Detailed qualitative analysis results were provided in the Supplementary Materials (Appendix E2).

### Quantitative image analysis

Quantitative assessment results are summarized in Table 3. There were significant differences in CT numbers among different groups (all *p* < 0.001). ULDCT EDM revealed significantly lower noise in fat, aorta, and muscle than other reconstructions (*p* < 0.001 for all comparisons). Compared with RDCT HIR, ULDCT EDM showed significantly higher SNR (44.0 ± 77.2 vs. 4.6 ± 6.6; *p* < 0.001) and CNR (26.7 ± 17.7 vs. 5.0 ± 4.4; *p* < 0.001) for pulmonary nodules. Furthermore, VMIs at 40 keV and 70 keV of ULDCT yielded lower noise and higher SNR than ULDCT HIR, although the differences were not statistically significant (*p* > 0.05 for all).

**Table 3:** Quantitative image quality analysis results

	RDCT	ULDCT				*p-*value^a^	*p*-value^b^			
		HIR	EDM	VMI_40keV_	VMI_70keV_		RDCT vs. HIR	RDCT vs. EDM	RDCT vs. VMI_40keV_	RDCT vs. VMI_70keV_
CT number (HU)										
All nodules	−315.1 ± 300.0	−306.7 ± 290.0	55.0 ± 23.8	−257.7 ± 354.5	−387.3 ± 272.5	< 0.001	1.000	< 0.001	0.018	< 0.001
Solid	−83.7 ± 196.0	−104.9 ± 207.8	69.9 ± 20.1	−19.2 ± 263.4	−210.4 ± 206.3	< 0.001	1.000	< 0.001	0.001	< 0.001
Part-solid	−364.2 ± 139.8	−349.5 ± 140.7	54.6 ± 11.7	−270.1 ± 196.2	−394.6 ± 149.2	< 0.001	1.000	< 0.001	0.159	1.000
Pure GGN	−604.7 ± 116.1	−586.1 ± 129.4	33.7 ± 10.3	−597.8 ± 154.8	−639.8 ± 141.1	< 0.0001	1.000	< 0.001	1.000	0.069
Fat	−103.0 ± 18.4	−106.4 ± 25.8	93.5 ± 1.9	−157.3 ± 38.5	−92.3 ± 46.3	< 0.0001	0.954	< 0.001	< 0.001	< 0.001
Aorta	48.6 ± 33.6	55.2 ± 26.5	103.5 ± 2.0	80.7 ± 44.0	48.4 ± 18.2	< .0001	0.004	< 0.001	< .001	1.000
Muscle	48.7 ± 15.8	51.0 ± 28.0	100.9 ± 5.5	173.6 ± 164.2	44.4 ± 28.1	< 0.001	1.000	< 0.001	< 0.001	1.000
Image noise (HU)										
Fat	40.7 ± 24.0	160.9 ± 49.5	0.4 ± 0.4	152.6 ± 39.5	154.8 ± 61.6	< 0.001	< 0.001	< 0.001	< 0.001	< 0.001
Aorta	60.6 ± 38.1	251.0 ± 75.3	0.5 ± 0.4	238.0 ± 55.1	236.7 ± 55.0	< 0.001	< 0.001	< 0.001	< 0.001	< 0.001
Muscle	65.1 ± 39.0	227.7 ± 68.2	0.9 ± 0.9	219.9 ± 51.8	215.1 ± 51.1	< 0.001	< 0.001	< 0.001	< 0.001	< 0.001
SNR	4.6 ± 6.6	2.0 ± 2.1	44.0 ± 77.2	2.3 ± 2.2	2.7 ± 2.4	< 0.001	< 0.001	< 0.001	< 0.001	< 0.001
CNR	5.00 ± 4.4	0.8 ± 0.7	26.7 ± 17.7	1.1 ± 0.3	0.6 ± 0.2	< 0.001	< 0.001	< 0.001	< 0.001	< 0.001

The values are presented as the mean ± the standard deviation

*RDCT* regular-dose CT, *ULDCT* ultra-low dose CT, *HIR* hybrid iterative reconstruction, *EDM* electron density map, *VMI* virtual monoenergetic image, *GGN* ground-glass nodule, *SNR* signal-to-noise ratio, *CNR* contrast-to-noise ratio

The *p*-values^a^ were calculated using a repeated one-way analysis of variance (ANOVA). A value of *p* < 0.05 indicates a statistically significant difference. The *p*-values^b^ were calculated using the Bonferroni post hoc test. A value of *p* < 0.0125 (0.05/4) indicates a statistically significant difference

### Diagnostic performance

Nodule detection rates were given in Table 4, categorized by nodule type, reconstructions, and Lung-RADS category. Compared with the reference standard, reconstruction techniques of ULDCT images performed good detection of solid nodules, with a similar nodule detection rate (78.3%–82.6%). Among all reconstructions, only 1 of 35 part-solid nodules, sizing 10 mm, was missed on VMI_70keV_. For pure GGNs, EDM showed a significantly higher nodule detection rate (85.5%, 200/234), compared with HIR (76.9%, 180/234), VMI_4__0keV_ (76.5%, 179/234), and VMI_70keV_ (71.8%, 168/234). On EDM, 34 pure GGNs not identified, and all of them were less-than 6 mm in diameter.

**Table 4:** The nodule detection rates at ULDCT according to the reference standard

Reconstructions	Nodule type	Lung-RADS category						Total
		1	2	3	4A	4B	4X	
ULDCT HIR	Solid	89.3% (50/56)	66.3% (106/160)	81.8% (90/110)	100% (30/30)	100% (7/7)	100% (5/5)	78.3% (288/368)
	Part-solid	/	/	100% (26/26)	/	100% (6/6)	100% (3/3)	100% (35/35)
	Pure GGN	/	76.9% (180/234)	/	/	/	/	76.9% (180/234)
ULDCT EDM	Solid	87.5% (49/56)	75.6% (121/160)	83.6% (92/110)	100% (30/30)	100% (7/7)	100% (5/5)	82.6% (304/368)
	part-solid	/	/	100% (26/26)	/	100% (6/6)	100% (3/3)	100% (35/35)
	pure GGN	/	85.5% (200/234)	/	/	/	/	85.5% (200/234)
ULDCT VMI_40keV_	solid	85.7% (48/56)	70.6% (113/160)	83.6% (92/110)	100% (30/30)	100% (7/7)	100% (5/5)	80.2% (295/368)
	Part-solid	/	/	100% (26/26)	/	100% (6/6)	100% (3/3)	100% (35/35)
	Pure GGN	/	76.5% (179/234)	/	/	/	/	76.5% (179/234)
ULDCT VMI_70keV_	Solid	85.7% (48/56)	70.6% (113/160)	80.9% (89/110)	100% (30/30)	100% (7/7)	100% (5/5)	79.3% (292/368)
	Part-solid	/	/	96.2% (25/26)	/	100% (6/6)	100% (3/3)	97.1% (34/35)
	Pure GGN	/	71.8% (168/234)	/	/	/	/	71.8% (168/234)

*Lung-RADS* Lung Imaging Reporting and Data System, *ULDCT* ultra-low dose CT, *HIR* hybrid iterative reconstruction, *EDM* electron density map, *VMI* virtual monoenergetic image, *GGN* ground-glass nodule

The results of inter-scan agreement and inter-observer agreement were summarized in Table 5. Inter-scan agreements between ULDCT and SDCT were good (all κw > 0.60, both reader 1 and reader 2). The inter-observer agreements between the two readers were good for ULDCT HIR and ULDCT VMI_70keV_, and excellent for ULDCT EDM and ULDCT VMI_40keV_.

**Table 5:** Inter-scan and Inter-observer agreement for Lung-RADS results

Reconstructions	Inter-scan agreement		Inter-observer agreement
	Reader 1	Reader 2	
SDCT vs. ULDCT HIR	0.636	0.606	
SDCT vs. ULDCT EDM	0.755	0.754	
SDCT vs. ULDCT VMI_40keV_	0.750	0.742	
SDCT vs. ULDCT VMI_70keV_	0.663	0.661	
ULDCT HIR			0.740
ULDCT EDM			0.818
ULDCT VMI_40keV_			0.822
ULDCT VMI_70keV_			0.762

*SDCT* standard dose CT, *ULDCT* ultra-low dose CT, *HIR* hybrid iterative reconstruction, *EDM* electron density map, *VMI* virtual monoenergetic image, *GGN* ground-glass nodule

**Fig. 6:**
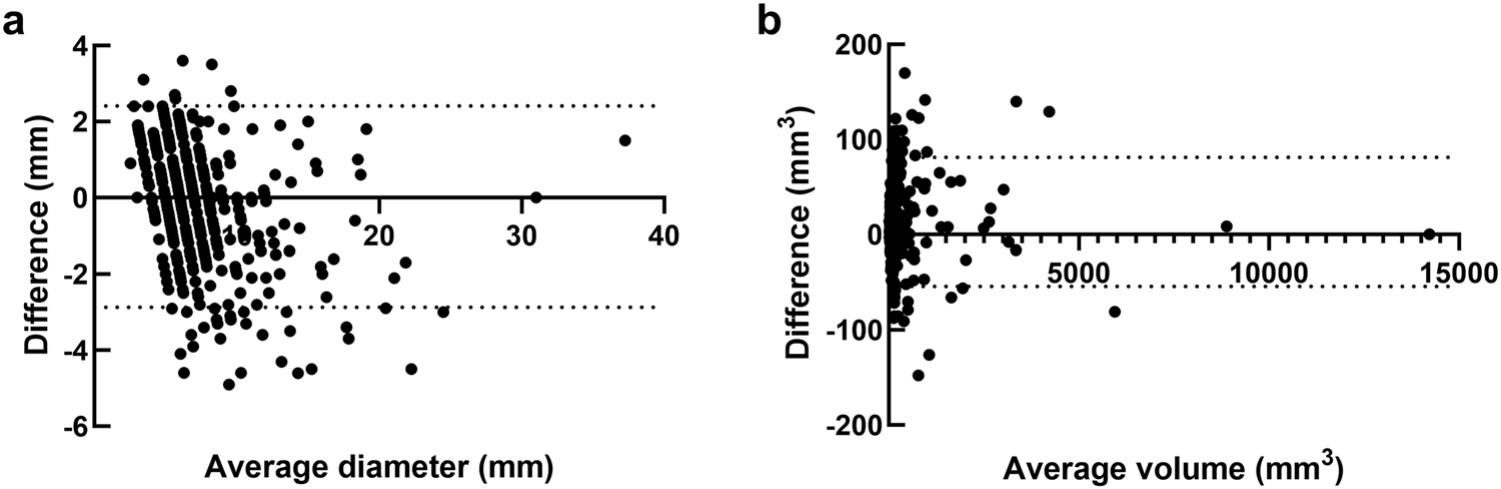
Bland-Altman plots show the consistency of diameter measurement (**a**) and volume measurement (**b**) between RDCT HIR and ULDCT EDM. RDCT, regular-dose CT; ULDCT, ultra-low dose CT; HIR, hybrid iterative reconstruction; EDM, electron density mapping

The consistency of measurement between RDCT and ULDCT was excellent for diameter ((ICC value) RDCT vs. ULDCT HIR: 0.907, RDCT vs. ULDCT EDM: 0.913, RDCT vs. ULDCT VMI_40keV_: 0.901, RDCT vs. ULDCT VMI_70keV_: 0.895) and volume ((ICC value) RDCT vs. ULDCT HIR: 0.977, RDCT vs. ULDCT EDM: 0.989, RDCT vs. ULDCT VMI_40keV_: 0.981, RDCT vs. ULDCT VMI_70keV_: 0.975). Consistency in nodule diameter and volume measurements between the two readers was excellent (all ICC values > 0.80). Figure 6 shows the Bland-Altman plots of RDCT HIR vs. ULDCT EDM for diameter and volume measurements. Subgroup analysis based on nodule type and size is provided in the Supplementary Materials (Appendix E3).

## Discussion

The incidental finding of pulmonary nodules is increasingly common in CT lung cancer screening. In our study, dual-layer spectral CT (DLSCT) at a mean effective radiation dose of ~0.3 mSv demonstrated satisfactory nodule identification and characterization. To the best of our knowledge, this was the first study to assess image quality and Lung Imaging Reporting and Data System (Lung-RADS) evaluation of lung nodules with ultra-low dose dual-energy CT. For qualitative analysis, electron density map (EDM) relative to virtual monochromatic images (VMIs) and conventional hybrid iterative reconstruction (HIR), showed greater overall quality with median subjective scores of good image quality, especially for GGNs. These results were confirmed by quantitative image quality analysis with a favorable nodule detection rate of 84.6% for EDM at ultra-low dose CT (ULDCT). Furthermore, there was a good agreement (the weighted kappa = 0.755) compared with the reference standard in the classification of pulmonary nodules. Altogether, these key findings provide further evidence that DLSCT is a good candidate for lung cancer screening or frequent follow-up for pulmonary nodules at dose reduction scans.

Based on the as-low-as-reasonably-achievable radiation principle, radiation exposure reduction has become increasingly important. Previous literature has shown that the combination of scanning protocol optimization and iterative reconstruction algorithms achieved radiation dose reduction without severely sacrificing image quality in a clinical setting [28–30], some results were reinforced by the findings of our study. In our work, the mean effective dose for 100 kV at ULDCT (0.3 mSv) was similar to or lower than the low dose CT reported by other study groups (0.29–0.93 mSv) [31, 32]. This was a reduction of about 91.2% compared with the 3.6 mSv in the regular-dose scan.

In the present study, the overall image quality score and the subjective noise of EDM at 100 kVp CT were rated as at least average but never unacceptable. This is not surprising because EDM can typically distinguish inherited electron density between pathological change and normal lung tissue to reflect the nature of the nodules. Previous studies have demonstrated that EDM has the capability to improve image quality and lesion conspicuity for the evaluation of lung subsolid nodules and reflect changes in the invasiveness of GGNs accurately [26, 33–35]. In concordance with a prior study [33], EDM derived from DLSCT had the highest signal-to-noise ratio for visualization of GGNs, compared to conventional iterative reconstruction algorithms and VMIs. Moreover, HIR has serious streak artifacts, which affect the observation of nodules and the overall image quality. This effect was greatly reduced in EDM (subjective grading score, 4.9 ± 0.4 vs. 2.9 ± 0.5, *p* < 0.001). Regarding the objective image analysis, we found that at ultra-low dose settings, signal-to-noise ratio and contrast-to-noise ratio were significantly improved using VMI_40keV_ than using HIR. Prior similar studies have revealed that VMIs at 40–50 keV exhibited higher lesion contrast [21–24], which is consistent with our work. These findings can be attributed to the fact that VMIs at low monoenergetic levels have the potential to avoid the hardening beam effect to optimize the lesion contrast [21].

The American College of Radiology has released the Lung-RADS, which is designed to standardize reporting and management recommendations for CT screening for lung cancer and facilitate prognostic surveillance. Accurate identification and measurements of lung nodules are important for Lung-RADS classification and management decisions. In addition, nodule size has been demonstrated to be linked with the probability of lung adenocarcinoma occurrence and invasiveness [36, 37]. Kim et al reported [38] that VMIs of dual-energy CT potentially improved the reproducibility of subsolid nodule measurement, which allowed lung cancer screening at a lower radiation dose (0.9–2.7 mGy). Another study on chest phantom found that 100 kVp/10 mAs scan protocol based on spectral CT could quantify the diameter and density of pulmonary nodules accurately [39]. Similarly, our study showed that RDCT and ULDCT EDM had good agreement in the measurement of diameter and volume (all ICC values > 0.80).

Several studies have indicated that the detection of pulmonary nodules conspicuity might be improved by the use of IR techniques [12–15]. We found that the visualization and detection rate of pulmonary nodules were better with EDM (84.6%) than those with HIR (79.0%) on ULDCT, which was mainly related to the increased contrast to normal lung areas, and can fulfill the clinical requirements for lung nodule detection. Notably, 17.4% solid nodules (64/368) and 14.5% pure GGNs (34/234) were missed on EDM at ultra-low dose scan in this work. Fortunately, the missed nodules were small in diameter (< 6 mm) and mostly classified as Lung-RADS category 1 or 2. According to the guideline published by the Fleischner Society, the clinical significance of these small-sized (< 6 mm) GGNs is limited in lung cancer screening [40], because of their extremely low risk of invasive adenocarcinoma.

Our study has several limitations. First, the sample size in this single-center study was relatively small, especially for part-solid nodules, which account for a very small proportion of pulmonary nodules. This may limit the generalizability of the results to other populations or clinical settings. Future multi-center studies with larger sample sizes are needed to substantiate our preliminary results. Second, only an unenhanced chest CT scan was performed for lung cancer screening in this work, although contrast-enhanced images can provide valuable information on vascularization and potential malignancy of atypical or complex lung nodules. And the majority of nodules lacked pathological confirmation. Without biopsies or other diagnostic confirmation methods, there remains uncertainty concerning the diagnostic accuracy of DLSCT in distinguishing malignant from benign nodules. Third, because different reconstruction methods have unique appearances, complete blinding was difficult during analysis. This could introduce unintentional bias in image interpretation, affecting the objectivity of the results. Fourth, ultra-low dose results were evaluated on DLSCT from one vendor. The influence of different CT systems and post-processing software should be identified. Further studies are needed to confirm that these results are applicable to other dual-energy CTs. Fifth, considering the clinical significance of primary pulmonary nodules and the accuracy of measurement, metastatic pulmonary nodules and calcified nodules were excluded from our study. However, this likely represents the patient population in the clinic, which may affect the generalizability of the results to other clinical settings. Finally, this modest radiation dose reduction was chosen because the lowest available tube voltage was 100 kVp set at DLSCT.

In conclusion, ultra-low dose DLSCT with a 91.2% dose reduction results in satisfactory image quality and accurate Lung-RADS evaluation, revealing great potential for lung cancer screening and management in daily clinical practice.

## Supplementary information

ELECTRONIC SUPPLEMENTARY MATERIAL

## Data Availability

The datasets used and/or analyzed during the current study are available from the corresponding author and the first author on reasonable request. The data are not publicly available due to privacy or ethical restrictions.

## Abbreviations

BMI: Body mass index
DLSCT: Dual-layer detector spectral CT
EDM: Electron density map
GGN: Ground-glass nodule
HIR: Hybrid iterative reconstruction
Lung-RADS: Lung Imaging Reporting and Data System
RDCT: Regular dose CT
ULDCT: Ultra-low dose CT
VMI: Virtual monoenergetic image
