# Supplementary material for: Ultra-low dose dual-layer detector spectral CT for pulmonary nodule screening: image quality and diagnostic performance
Source: Insights Imaging. 2025 Jan 10;16:11. doi: 10.1186/s13244-024-01888-1 (PMC11723867; doi:10.1186/s13244-024-01888-1)
Supplement: Supplementary file 1 — ELECTRONIC SUPPLEMENTARY MATERIAL [file 13244_2024_1888_MOESM1_ESM.pdf]

Ultra-low dose dual-layer detector spectral CT for pulmonary nodule screening: Image quality and diagnostic performance

ELECTRONIC SUPPLEMENTARY MATERIAL

Appendix E1: CT scanning parameters

Each patient underwent an unenhanced regular dose CT (RDCT) scan first, followed by an ultra-low dose CT (ULDCT) scan. A 5 s interval was set between these two scans. The RDCT was obtained using a routine clinical protocol with automatic tube current modulation (DoseRight, Philips Healthcare) and a tube voltage of 120 kVp. ULDCT was performed with a fixed tube current of 10 mA and a tube voltage of 100 kVp. The remaining scanning parameters for RDCT and ULDCT were identical: 0.27-second gantry rotation time, pitch of 1.376, and 64 detector row × 0.625 mm detector configuration. All CT examinations were acquired in the craniocaudal direction in the supine position.

Appendix E2: Qualitative Image Analysis

Qualitative assessment results were summarized in Table S1. In terms of streak artifact, EDM achieved the highest score compared to other reconstructions (P < .001). Concerning image noise on ULDCT, EDM achieved the highest score, followed by $VMI_{70\ keV}$, $VMI_{40\ keV}$ and then HIR. Regarding clarity of small vessels on ULDCT, EDM was rated significantly better than the others (all P < .001). Regarding overall image quality for ultra-low dose images, HIR demonstrated the lowest score (2.9 ± 0.6).

Appendix E3: Subgroup analysis

Table S2 summarized the diameter and volume measured by different reconstruction algorithms. A subgroup analysis based on nodule type revealed that the ICC value for the diameter of pure ground glass, part-solid, and solid nodules was 0.822,0.930, and 0.951, respectively. The ICC value for the volume of pure ground-glass, part-solid, and solid nodules was 0.905, 0.975, and 0.990, respectively. A subgroup analysis based on nodule diameter found that the ICC value for inter-observer agreement on the diameter of < 5 mm, 5-10 mm, and >10 mm was 0.432, 0.753, and 0.901, respectively. The ICC value for inter-observer agreement on the volume of < 5 mm, 5-10 mm, and >10 mm was 0.502, 0.723, and 0.976, respectively. The Bland-Altman analysis between RDCT and ULDCT are shown in Table S3.

Table S1 Qualitative image quality analysis results

Reconstruction	streak artifact	image noise	visibility of small vessels	visibility of pulmonary nodules	overall image quality
Score (mean ± SD)					
RDCT	4.2±0.4	4.5±0.5	4.8±0.4	4.4±0.8	4.6±0.5
ULDCT					
HIR	2.9±0.5	2.7±0.7	3.3±0.8	3.6±1.3	2.9±0.6
EDM	4.9±0.4	3.8±0.5	3.7±0.9	4.3±0.8	3.8±0.7
$VMI_{40\ keV}$	3.0±0.6	2.8±0.7	3.6±0.7	3.8±1.1	3.1±0.6
$VMI_{70\ keV}$	3.1±0.6	2.9±0.6	3.1±0.7	3.4±1.2	3.0±0.7
P values					
RDCT vs HIR	＜0.001	＜0.001	＜0.001	＜0.001	＜0.001
RDCT vs EDM	＜0.001	＜0.001	＜0.001	1.000	＜0.001
RDCT vs 40 keV	＜0.001	＜0.001	＜0.001	＜0.001	＜0.001

RDCT vs 70 keV	＜0.001	＜0.001	＜0.001	＜0.001	＜0.001

Data are presented as the mean ± the standard deviation.

RDCT, regular dose CT; ULDCT, ultra-lowdose CT; HIR, hybrid iterative reconstruction; EDM, electron density map; VMI, virtual monoenergetic image.

The p values were calculated using post hoc Wilcoxon signed-rank test. A value of $P < 0.0125$ (0.05/4) indicates a statistically significant difference.

Table S2 Diameter and volume measured by different reconstruction algorithms

	RDCT	ULDCT				F value	*P* value [a]
		HIR	EDM	$VMI_{40\ keV}$	$VMI_{70\ keV}$		
Diameter (mm)	7.0 ± 3.3	7.4 ± 3.5	7.2 ± 3.7	7.4 ± 3.7	7.4 ± 3.7	0.69	0.631
Volume (mm^3)	254.1 ± 847.3	215.9 ± 627.2	245.4 ± 863.2	225.8 ± 651.3	218.3 ± 626.9	0.269	0.930

Data are presented as the mean ± the standard deviation. The *P* values [a] were calculated using a repeated one-way analysis of variance (ANOVA). A value of $P < 0.05$ indicates a statistically significant difference.

RDCT, regular dose CT; ULDCT, ultra-low dose CT; HIR, hybrid iterative reconstruction; EDM, electron density map; VMI, virtual monoenergetic image.

Table S3 The Bland-Altman analysis between RDCT and ULDCT

	Diameter measurement (%)	Volume measurement (%)
RDCT vs. HIR	-0.33 ± 1.34	21.95 ± 112.30
RDCT vs. EDM	-0.23 ± 1.35	13.65 ± 34.51
RDCT vs. $VMI_{40\ keV}$	-0.33 ± 1.44	12.16 ± 100.70
RDCT vs. $VMI_{70\ keV}$	-0.30 ± 1.51	21.75 ± 118.60

Data are described as % (difference/average) ± standard deviation.

RDCT, regular dose CT; ULDCT, ultra-low dose CT; HIR, hybrid iterative reconstruction; EDM, electron density map; VMI, virtual monoenergetic image.
